# Stromal Versican Accumulation and Proteolysis Regulate the Infiltration of CD8^+^ T Cells in Breast Cancer

**DOI:** 10.3390/cancers17091435

**Published:** 2025-04-25

**Authors:** Philip B. Emmerich, Tonela Qyli, Katherine A. Johnson, Somak Chaudhuri, Kristen M. Clark, Nathaniel B. Verhagen, Mitchell G. Depke, Linda Clipson, Cheri A. Pasch, Athanasios Papadas, Mark E. Burkard, Kari B. Wisinski, Stephanie M. McGregor, Fotis Asimakopoulos, Dustin A. Deming

**Affiliations:** 1 Division of Hematology and Oncology, Department of Medicine, University of Wisconsin-Madison, Madison, WI 53705, USA; 2 Cellular and Molecular Pathology Graduate Program, Department of Pathology and Laboratory Medicine, University of Wisconsin-Madison, Madison, WI 53705, USA; 3 McArdle Laboratory for Cancer Research, Department of Oncology, University of Wisconsin-Madison, Madison, WI 53705, USA; 4 University of Wisconsin Carbone Cancer Center, University of Wisconsin-Madison, Madison, WI 53705, USA; 5 Division of Blood and Marrow Transplantation and Moores Cancer Center, University of California-San Diego, La Jolla, CA 92093, USA; 6 Department of Pathology and Laboratory Medicine, University of Wisconsin-Madison, Madison, WI 53705, USA

**Keywords:** breast cancer, versican, CD8^+^ TILs, versikine, estrogen receptor

## Abstract

**Background/Objectives:** Recent clinical trials in breast cancer have demonstrated that some patients benefit from immune checkpoint blockade, though better predictive markers are needed. The accumulation of the immunomodulatory matrix proteoglycan versican (VCAN) can predict the exclusion of CD8^+^ tumor-infiltrating lymphocytes (TILs) in some settings and, thus, is evaluated in breast cancer here. **Methods:** A total of 230 breast cancers were analyzed for VCAN accumulation, VCAN proteolysis, and CD8^+^ TILs. CD8^+^ TILs were categorized based on their localization in the tumor epithelial or stromal compartments. **Results:** VCAN accumulation was detected in 90% of breast cancers, more commonly in ER+ tumors (93% vs. 77%; *p* < 0.001). MCF7 cells treated with estrogen upregulate VCAN without an enhanced expression of ADAMTS-proteases. VCAN-undetectable tumors demonstrate greater CD8^+^ TILs compared to VCAN-detectable tumors (*p* = 0.012). CD8^+^ T cells within TNBC tumors with high VCAN proteolysis infiltrated the epithelial compartment more often than in tumors with low VCAN proteolysis (91% vs. 42% respectively; *p* = 0.008). In the TCGA cohort, a strong inverse correlation between CD8A and VCAN expression was observed across subtypes. **Conclusions:** VCAN accumulation correlates with the exclusion of CD8^+^ TILs across subtypes of breast cancer, warranting further validation of VCAN accumulation and proteolysis as predictive biomarkers for breast cancer immunotherapy.

## Simple Summary

While clinical trials for immunotherapy in breast cancer have been promising, better markers that predict which patients may respond are needed. Versican is a protein abundant in many cancers that has been associated with the infiltration of CD8^+^ tumor-infiltrating lymphocytes. In this study, we aim to investigate the potential for the accumulation and proteolysis of versican to act as a biomarker for immunotherapy response in breast cancer by evaluating the relationship between versican, its proteolysis, and the infiltration of CD8^+^ tumor-infiltrating lymphocytes. We find a strong inverse correlation between versican accumulation and lymphocyte infiltration, warranting further investigation into versican as a potential biomarker of immunotherapy response in breast cancer.

## 1. Introduction

Despite advances in targeted therapies, breast cancer remains a leading cause of cancer-related death in women [1]. While breast cancer was initially thought to be resistant to immunotherapeutic agents, recent studies have shown some patients with triple-negative breast cancers (TNBC) are responsive to immunotherapy. The KEYNOTE-355 phase III clinical trial evaluated pembrolizumab plus chemotherapy versus (vs.) chemotherapy alone for previously untreated locally recurrent inoperable or metastatic triple-negative breast cancer [2]. In the intention-to-treat population, the median overall survival (mOS) was 17.2 months in the pembrolizumab-containing arm and 15.5 months with standard of care. In those patients with a combined positive score (CPS) of 10 or more, the pembrolizumab-containing arm had a mOS of 23 months compared to 16.1 months with control (HR 0.73, *p* = 0.0185) [3]. In the KEYNOTE-522 phase III study, patients with previously untreated stage II or III TNBC were randomized to neoadjuvant pembrolizumab and chemotherapy vs. placebo and chemotherapy [4]. A significant improvement in the pathologic complete response rate was observed with pembrolizumab treatment. Beyond TNBC, improved pathologic outcomes with neoadjuvant immunotherapy and chemotherapy have been observed in patients with estrogen receptor-positive and HER2-negative BC as part of the CheckMate-7FL and KEYNOTE-756 clinical trials [5,6].

The breast cancer tumor microenvironment (TME), as with many solid tumors, is a dynamic factor of the disease consisting of both protumor and antitumor components. Lymphocytes, tumor-associated macrophages and dendritic cells are well-characterized components of tumor-immune infiltrates [7]. In fact, CD8^+^ T cell infiltration has been shown to predict clinical benefit for breast cancer patients independent of many clinical factors, such as tumor grade and lymph node status [8]. Cancer-associated fibroblasts are derived from several cell types, including normal breast fibroblasts and recruited bone marrow cells [9,10] and contribute to the acellular component of the TME known as the extracellular matrix (ECM).

Major components of the breast cancer ECM include collagen, fibronectin, laminin, hyaluronic acid, and proteoglycans, such as versican (VCAN) [11]. The ECM in breast cancer promotes cancer cell invasion and proliferation while also creating a microenvironment that nurtures cancer progression [7] and establishes the metastatic niche by driving the process of the mesenchymal-to-epithelial transition [12]. The ECM has also been shown to partially regulate the immune microenvironment seen in breast cancer [13], such as the deposition of SPARC leading to an infiltration of regulatory T lymphocytes and myeloid-derived suppressor cells [14]. Additionally, more recent studies characterizing the stromal response in breast cancers have demonstrated a distinct correlation between fibroblast signature genes and cytotoxic T-cell dysfunction and exclusion [15].

We recently discovered that an ECM immunomodulatory proteoglycan versican (VCAN) and its ADAMTS-protease proteolytic cleavage product versikine (Vkine) differentially accumulate in the TME of multiple myeloma [16] and colorectal cancers (CRC) [17]. In CRC, increased VCAN accumulation was associated with decreased CD8^+^ TILs, likely through dendritic cell dysfunction [17]. Conversely, CRC tumors with high VCAN proteolysis (low total VCAN and high cleaved VCAN staining) had a 10-fold increase of CD8^+^ tumor-infiltrating lymphocytes (TILs) over tumors with lower VCAN proteolysis. In breast cancer, VCAN production is partially driven by cancer-associated fibroblasts under the influence of cancer cells [18], potentially through TGF-β signaling [19]. It has also been recently demonstrated that the accumulation of VCAN is strongly associated with an influx of tumor-associated macrophages, an immunosuppressive immune cell population [20]. VCAN has been shown to have prognostic value in breast cancer, as patients with elevated VCAN expression demonstrated a higher relapse rate and reduced overall survival rates [18,21]. However, the correlation between VCAN accumulation and tumor-infiltrating CD8^+^ T cells in breast cancer has been understudied and is the primary focus of this investigation. We hypothesize that the presence of VCAN in the stromal compartment will limit CD8^+^ T cell infiltration, and its proteolysis will be associated with enhanced infiltration.

## 2. Materials and Methods

### 2.1. Breast Cancer Patient Specimens

All study procedures were completed per a University of Wisconsin–Madison Health Sciences IRB-approved protocol (IRB# 2016-0934). The research herein conforms to the principles outlined in the Declaration of Helsinki. Waiver of consent was granted by the University of Wisconsin–Madison. A total of 371 cases of invasive breast cancer were identified and used to generate a tissue microarray (TMA) that was obtained through the UW–Madison Translational Science Biocore [22]. Cases without invasive carcinoma, as well as cases with poor quality or exclusively stromal content on the IHC-stained slides, were excluded from our studies, leaving a total of 230 cases in our final cohort. Clinical data were collected for this patient cohort, including age, histologic subtype, stage, receptor status, date of diagnosis, race, and menopausal status. Estrogen receptor (ER) and progesterone receptor (PR) statuses were reported as positive or negative and also provided in the form of the Immunoreactive Remmele Score (IRS) [23].

### 2.2. Immunohistochemistry (IHC)

Unstained TMA tissue sections were deparaffinized and rehydrated according to standard procedures. Antigen retrieval was conducted using citrate buffer (pH 6.0) for αDPEAAE (VCAN proteolysis) staining, EDTA buffer (pH 8.0) for CD8 staining, and chondroitinase ABC for VCAN staining as previously described [24]. The following primary antibodies were used: VCAN (1:100; 12C5; Developmental Studies Hybridoma Bank; University of Iowa, Iowa City, IA, USA), αDPEAAE (1:2000; PA1-1748; Fisher Scientific; Hampton, NH, USA), CD8a (1:200; c4-0085-80, eBioscience; San Diego, CA, USA). Betazoid DAB buffer and chromogen (SKU DS900, BioCare Medical, Pacheco, CA, USA) were used to visualize staining, and hematoxylin was used for nucleic acid counterstain.

### 2.3. IHC Quantification

An Olympus BX40 microscope (Olympus; Waltham, MA, USA) was used to visualize all stained slides. To quantify VCAN and αDPEAAE staining, a binning system was utilized based on the intensity of stromal staining only. Each core was assigned a score between 0 and 3 (0 = no staining; 1 = light and minimal staining; 2 = moderate intensity, may be patchy areas of strong intensity; 3 = intense staining throughout the majority of stroma). Three different systems were used to quantify CD8^+^ T cells throughout this study to reflect different approaches within the field of breast cancer: total, epithelial, and stromal lymphocytes. For each of these systems, CD8^+^ lymphocytes were counted within a 400× high-powered field of view (HPF). Total CD8^+^ T cell values were compiled by manually counting lymphocytes, which demonstrated positive CD8 staining, regardless of localization within tumor tissue. Epithelial counts were compiled by counting CD8^+^ lymphocytes in direct contact with breast cancer cells based on histology. Stromal CD8^+^ T cell counts were compiled by counting CD8^+^ lymphocytes, which were within the stromal compartment of the tissue and not in contact with breast cancer cells. For both epithelial and stromal quantification, HPFs within each core were selected for the greatest proportion of epithelial and stromal content, respectively; therefore, it would not be expected that the epithelial and stromal CD8^+^ TILs would equal that of the total number of reported CD8^+^ TILs. To verify the consistency of the immune infiltration on cores represented on the TMA, 32 full tissue slides were obtained from patients, and epithelial CD8 T cell counts averaged across five HPFs were compared to the TMA cores.

### 2.4. Estrogen Stimulation

The ER+ MCF7 cells were acquired as a gift from the lab of Elaine Alarid, PhD (McArdle Laboratory, Madison, WI, USA). One hundred thousand cells were plated in stripping media [Phenol-red free DMEM (Gibco, Grand Island, NY, USA) supplemented with 10% charcoal-stripped FBS (Gibco, REF# A33821-10, Grand Island, NY, USA), 2% Glutamax (Grand Island, NY, USA), 1% Pen Strep (Gibco, Grand Island, NY, USA), 1% sodium pyruvate (Grand Island, NY, USA)] in each well across two six-well tissue-culture treated plates. Ninety-six hours later, the media was replaced with either stripping media with 10 nM β-Estradiol (1:1000 10 µM in media + 1% ethanol) (Sigma-Aldrich, St. Louis, MO, USA) or vehicle control. Controls were collected 10 h following administration of vehicle control. Cells were collected at the indicated time points by rinsing in DEPC-treated 1× PBS. Results represent four biological replicates conducted across two independent experiments.

### 2.5. qPCR

RNA was isolated using the RNeasy Plus Mini Kit (Qiagen, Hilden, Germany) and 40 ng of RNA was converted to cDNA using GoScript™ Reverse Transcriptase (Promega, Madison, WI, USA). qPCR was conducted in a 96-well format using the SensiFAST™ SYBR^®^ No-ROX Kit (Thomas Scientific, Swedesboro, NJ, USA) and read on a BioRad© CX96 Touch Real-Time PCR Detection System (BioRad, Hercules, CA, USA). Primers were ordered through IDT™ (Coralville, IA, USA) and are as follows: PGR (Forward, 5′-GCTGTCATTATGGTGTCCTTAC-3′: Reverse, 5′-GTAGTTGTGCTGCCCTTCC-3′), TFF1 (Forward, 5′-GTCCCCTGGTGCTTCTATCCT-3′: Reverse, 5′-AGCCGAGCTCTGGGACTAATC-3′)) VCAN (Forward, 5′-GAGGTGGTCTACTTGGGGTGA-3′: Reverse, 5′-ACAAGTGGCTCCATTACGAC-3′) ADAMTS1 (Forward, 5′-GCACTGCAAGGCGTAGGAC-3′: Reverse, 5′-AACGATGGTTTCCACATAGCG-3′), ADAMTS4 (Forward, 5′-GGGATAGTGACCACATTGTT-3′: Reverse, 5′-AGGCACTGGGCTACTACTAT-3′) ADAMTS5 (Forward, 5′-CACTGTGGCTCACGAAATCG-3′: Reverse, 5′-CGCTTATCTTCTGTGGAACCAAA-3′), GAPDH (Forward 5′-AATCCCATCACCATCTTCCA-3′: Reverse, 5′-TGGACTCCACGACGTACTCA-3′). The relative expression values were normalized to the housekeeping gene GAPDH, followed by normalizing to the control timepoint.

### 2.6. Gene Expression Profiling

The cBioPortal was utilized as previously described to acquire full gene expression analyses [25,26]. Briefly, the publicly available dataset for breast invasive carcinoma (TCGA, PanCancer Atlas) was analyzed according to subtype (Luminal A, Luminal B, basal [TNBC], and HER2-enriched). Subsequently, a gene query was conducted using the genes indicated in each figure. Heat maps are visual representations for mRNA expression z-scores relative to diploid samples (RNA Seq V2 RSEM) and demonstrate the data upon clustering for visual effect. Heatmaps were made using ComplexHeatmap (version 2.16.0 [27,28]) in R (version 4.3.0 [29]).

### 2.7. Versican Proteolytic Phenotype Signatures and Gene Set Enrichment Analysis

A cohort of 994 patient samples from the Breast Invasive Carcinoma TCGA PanCancer Atlas dataset [30] was chosen to identify Versican Proteolysis Predominant (VPP) and Versican Proteolysis Weak (VPW) subsets of breast cancer. mRNA z-score cutoffs for high and low expression of VCAN, ADAMTS4, or TIMP3 were set as >0.5 or <−1, respectively, to define VPP (VCAN^hi^ ADAMTS4^hi^ TIMP3^intermediate/low^, *n* = 48) and VPW (VCAN^hi^ ADAMTS4^intermediate/low^ TIMP3^hi^, *n* = 66) groups. Group differences in genomic alteration frequencies, disease staging, MSI MANTIS scores, tumor mutation burden, median diagnosis age, sex/race distribution, and relative expression of immunologically interesting markers were analyzed via cbioportal.org [25,26,31]. Differential expression data comparing VPP and VPW groups were used for Gene Set Enrichment Analysis (GSEA) [32,33] against the MSigDB Hallmark gene sets [34]. The differential expression of genes within gene sets was analyzed using R (Version 4.3.0 [29]), and all graphs were plotted using Graph Prism (Version 7.0a, 2 April 2016).

### 2.8. Statistical Considerations

Wilcoxon rank sum, *t*-tests, Chi-squared, and Jonckheere trend tests were conducted where reported in this manuscript. For all figures, * indicates *p* ≤ 0.05, ** indicates *p* ≤ 0.01, and *** indicates *p* ≤ 0.001.

## 3. Results

### 3.1. Clinical Characteristics

A representative cohort of patients at the University of Wisconsin Carbone Cancer Center was selected for this study. Most patients were white (*n* = 220), with the remaining either black (*n* = 5), Asian (*n* = 2), Hispanic (*n* = 2), or of unknown descent (*n* = 1). All patients were females between the ages of 30 and 94 at diagnosis. Most patients did not have a family history of breast cancer (*n* = 188), though a portion did (*n* = 37) with five patients of unknown family history. The majority of patients were post-menopausal (*n* = 140), though many were pre-menopausal (*n* = 86), perimenopausal (*n* = 1), or unknown (*n* = 3). Patients varied across stage (Stage I, *n* = 94; Stage II, *n* = 106; Stage III, *n* = 30) and tumor grade (grade 1, *n* = 41; grade 2, *n* = 106; grade 3, *n* = 83). Additionally, patients were divided into four distinct categories according to the hormone receptor and HER2 status of their tumors, including ER+/PR+/HER2− (*n* = 157), ER+/PR+/HER2+ (*n* = 25), ER−/PR−/HER2+ (*n* = 11), and ER−/PR−/HER2−, or triple-negative breast cancer (TNBC; *n* = 37).

### 3.2. VCAN Accumulation Is a Common Feature of Breast Adenocarcinoma

**Figure 1:**
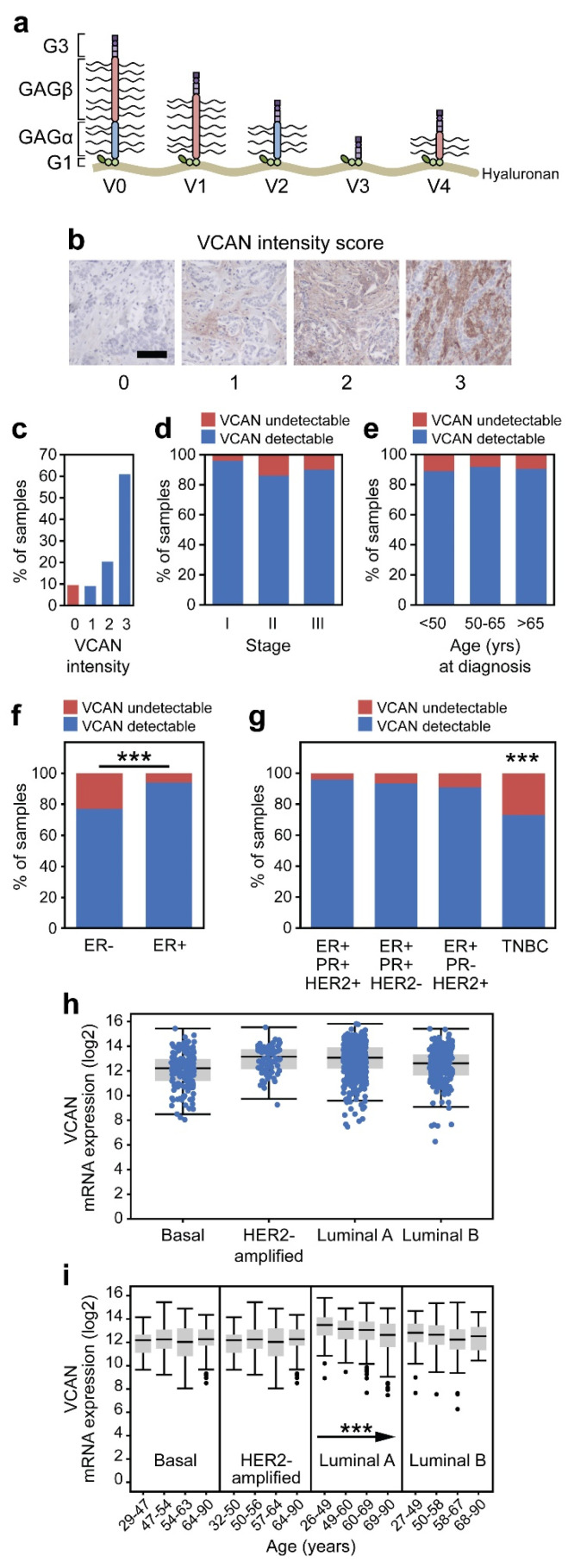
VCAN accumulation across breast cancers. (**a**) VCAN is expressed as one of five isoforms in breast cancer due to alternative splicing, each of which is recognized via IHC due to antibody binding of the conserved G1 domain. (**b**) Representative images of VCAN binning intensities. The scale bar is 100 µm. (**c**) Patients were grouped based on stromal intensity across all cancers and subsequently separated into VCAN-undetectable (intensity of 0) or VCAN-detectable (intensity of 1–3). (**d**–**g**) The distribution of VCAN-detectable tumors is represented across patients of different stages, age ranges, ER status and subsequent ER/PR/HER2 status (Chi-square). VCAN expression as a function of subtype (**h**) and age within each subtype ((**i**) Jonckheere trend test) from TCGA data. *** *p* < 0.001.

Versican (VCAN) is commonly found within the ECM of solid tumors, anchored to hyaluronan through a pair of binding modules in the G1 domain (Figure 1a). Five main isoforms of VCAN exist as a product of alternative splicing of exons 7 and 8, which correspond to the GAGα and GAGβ domains, respectively, found in the central chondroitin-sulfate binding domain of the molecule [35]. The C-terminal G3 domain consists of two epidermal growth factor (EGF)-like repeats, a lectin-like motif (carbohydrate recognition domain), and a complement binding protein (CBP)-motif. The antigenic portion of VCAN recognized by the antibody is within the G1 domain and is therefore conserved across each of the isoforms [36]. A binning system was used to quantify the intensity of VCAN within the stroma (score = 0–3). (Figure 1b). VCAN accumulation within the stroma was abundant (score = 2–3) in 81.3% of breast cancers (Figure 1c). The proportion of cancers with detectable VCAN (1–3) did not significantly vary across the stage of the disease (Figure 1d) or the age of the patient (Figure 1e).

Breast cancer is a heterogeneous disease that has classically been grouped based on the expression of estrogen receptor (ER) and progesterone receptor (PR) as well as the overexpression of human epidermal growth factor receptor 2 (HER2) in tumors. Of the cancers that were investigated, 80% were ER-positive (ER+) (*n* = 184), and 20% were ER-negative (ER−) (*n* = 46). VCAN-detectable tumors represented a larger proportion of ER+ cancers than ER− cancers (93% vs. 77%; *p* < 0.001; Figure 1f). Twenty-seven percent of TNBCs lacked VCAN accumulation—the highest rate among subtypes (ER+/PR+/HER2+ 4%, ER^+^/PR^+^/HER2− 7%, HER2+ 9.1%, TNBC 27%; *p* < 0.001; Figure 1g).

Next, the expression of VCAN was assessed across the clinical subtypes of breast cancer using the TCGA dataset. The samples were divided across Luminal A (hormone-receptor-positive, HER2 negative, low Ki67), Luminal B (hormone-receptor-positive, HER2-negative or positive, high Ki-67), HER2-enriched/amplified (hormone-receptor negative, HER2-positive), and basal/triple negative (hormone-receptor negative, HER2 negative). Variable levels of VCAN expression were seen within the entire cohort but did not vary significantly between the clinical subtypes (Figure 1h). Interestingly, it was found that the expression of VCAN decreases as a function of age within the Luminal A subtype (*p* < 0.001; Figure 1i), potentially related to the presence of estrogen.

### 3.3. Estrogen Stimulation of VCAN Expression

**Figure 2:**
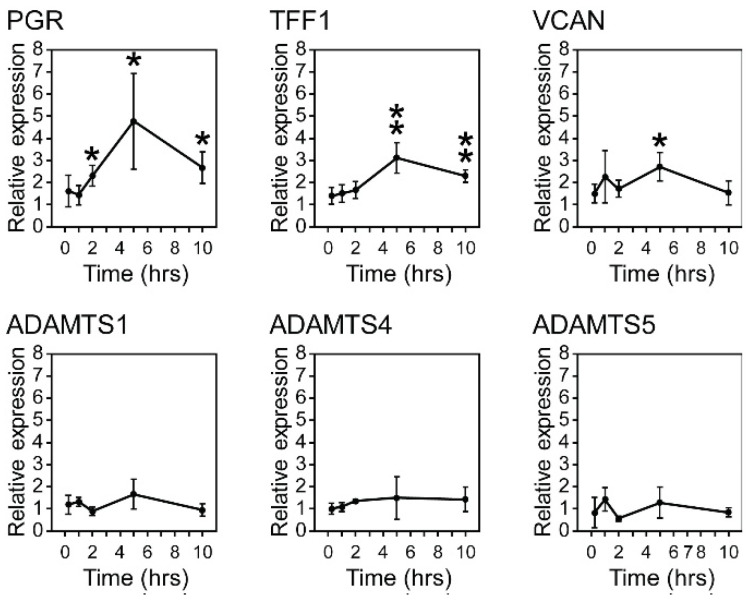
Estrogen receptor signaling increases VCAN expression in ER+ breast cancer cells. MCF7 cells were cultured for 96 h in DMEM + 10% charcoal-stripped FBS prior to the addition of 10 nm β-estradiol or vehicle control. qPCR was conducted to determine the expression of known estrogen response genes PGR and TFF1 as well as VCAN and ADAMTS1, 4, and 5. RNA was harvested from cells collected at the indicated time point following treatment and compared to vehicle-control cells collected at 10 h (*t*-test). * *p* < 0.05; ** *p* < 0.01.

As our findings demonstrated a significantly higher percentage of VCAN-detectable tumors in ER+ cancers compared to ER− cancers, there exists the possibility of a link between estrogen signaling and VCAN accumulation. To investigate this further, ER+ MCF7 cells were stimulated over 10 h with 10 nM β-estradiol and compared at various time points (15 min, 1 h, 2 h, 5 h, and 10 h) to a vehicle-only control (Figure 2). Relative expression levels of the estrogen-response genes progesterone receptor (PGR) and trefoil factor 1 (TFF1) served as positive controls. Estrogen response was identified at two hours (PGR only), as well as at five and ten hours. VCAN expression was significantly upregulated at five hours (2.7-fold increase, *p* = 0.013). However, none of the ADAMTS proteases were enhanced under estrogen stimulation, showing that the enhanced expression of VCAN is not coupled with increased cell-autonomous proteolysis under estrogen stimulation in MCF7 cells.

### 3.4. VCAN Accumulation Predicts CD8^+^ T Cell Exclusion in Breast Cancer

**Figure 3:**
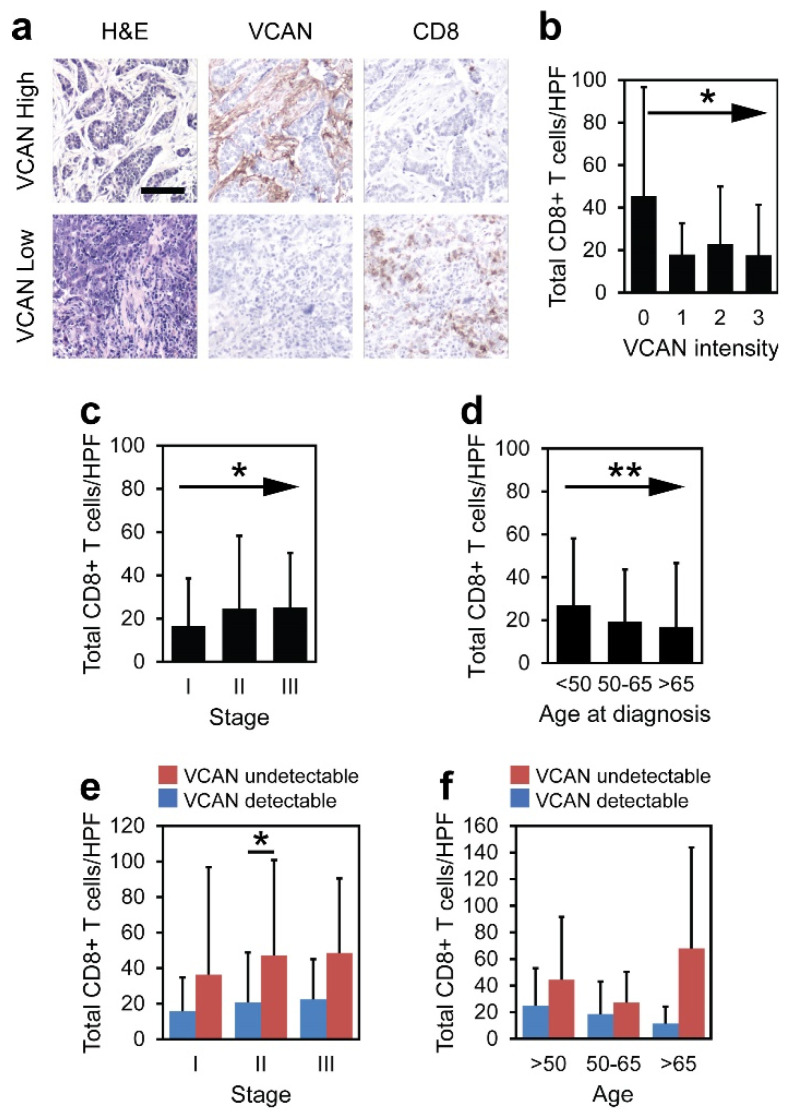
VCAN detection is associated with reduced presence of CD8^+^ T cells across ages and stages. (**a**) Representative images of staining of H&E, VCAN, and CD8^+^ TILs across examples of VCAN-detectable/undetectable samples. The scale bar is 100 µm. (**b**–**d**) Total CD8^+^ TILs across VCAN intensity, stage of disease, and age of the patient (Jonckheere trend test). (**e**,**f**) Total CD8^+^ TILs across the stage of disease and age of patient according to VCAN status (Wilcoxon rank sum). * *p* < 0.05; ** *p* < 0.01.

VCAN intensity was correlated with total CD8^+^ T cell numbers across patients (Figure 3a). Total CD8^+^ T cells for this study were quantified by counting the total number of positively stained lymphocytes per 400× high powered field (HPF), regardless of localization within the tissue. Cancers without detectable stromal VCAN staining had the greatest number of CD8^+^ T cells/HPF compared to those with detectable VCAN staining (*p* = 0.012; Figure 3b).

CD8^+^ T cells increased in more advanced stages of disease (*p* = 0.048; Figure 3c). Additionally, total CD8^+^ T cell numbers within tumors were reduced in older patients (*p* = 0.0028; Figure 3d). VCAN-undetectable tumors consistently contained more total CD8^+^ T cells than VCAN-detectable tumors across both age and stage of disease, reaching significance in Stage II (*p* = 0.029; Figure 3e,f). Many of the VCAN-undetectable subcategories had fewer than five patients, limiting statistical analyses. Total CD8^+^ T cells were also found to be similar in pre- and post-menopausal cancers (Supplementary Figure S1). Interestingly, post-menopausal status was associated with significantly more total CD8^+^ T cells in VCAN-undetectable cancers than in VCAN-detectable cancers (*p* < 0.001), but pre-menopausal status did not reach significance (Supplementary Figure S1).

### 3.5. VCAN Predicts CD8^+^ T Cells Across Clinical Subtypes of Breast Cancer

**Figure 4:**
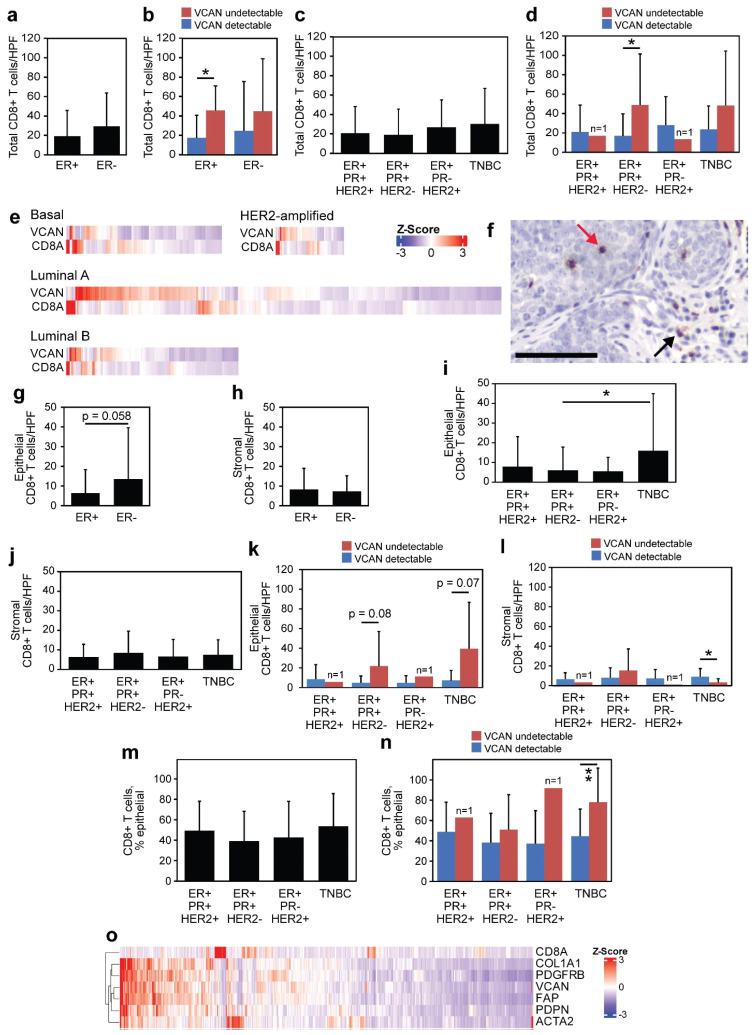
VCAN predicts CD8^+^ lymphocyte infiltration. (**a**,**b**) Total CD8^+^ TILs were assessed according to ER status alone (**a**) or in conjunction with VCAN status (**b**). (**c**,**d**) Similar assessments were conducted according to ER/PR/HER2 status. Statistical significance was determined using Wilcoxon rank-sum tests. (**e**) Gene-expression heatmaps clustered to show the relationship between CD8A and VCAN expression across breast cancer subtypes using TCGA data. (**f**) The localization of each CD8^+^ T cell was determined to be either epithelial (red arrow) or stromal (black arrow). The scale bar is 100 µm. (**g**,**h**) Epithelial and stromal CD8^+^ T cell numbers were assessed according to ER status. (**i**–**l**) Epithelial and stromal CD8^+^ T cell numbers were assessed across ER/PR/HER2 subgroups at baseline and according to VCAN status. (**m**,**n**) The epithelial % of CD8^+^ T cells was calculated as follows: (epithelial)/(epithelial + stromal) × 100. This was assessed across ER/PR/HER2 status and VCAN status. Statistical significance was determined using Wilcoxon rank-sum tests. (**o**) Gene expression analysis clustered to demonstrate associations between *CD8A*, *COL1A1*, *FAP*, *VCAN*, *PDGFRB*, *PDPN*, and *ACTA2* across the TCGA PanCancer breast cancer database. * *p* < 0.05; ** *p* < 0.01.

Next, we analyzed the total CD8^+^ T cells in these cancers according to receptor status. ER+ cancers demonstrated a strong trend towards fewer total CD8^+^ T cells as compared to ER− disease (*p* = 0.06; Figure 4a). Undetectable VCAN was associated with a significant increase in total CD8^+^ T cells in ER+ cancers (*p* = 0.028; Figure 4b). Upon including the PR and HER2 status of these cancers, it was found that TNBCs demonstrated the greatest average of total CD8^+^ T cells/HPF (Figure 4c). Interestingly, despite demonstrating the fewest total CD8^+^ T cells/HPF as a group, ER+/PR+/HER2− disease demonstrated a significant increase in total CD8^+^ T cells in VCAN-undetectable tumors (*p* = 0.032), and a similar trend was identified within TNBC (23.6 vs. 48.1, *p* = 0.3; Figure 4d).

Additionally, the TCGA dataset was used to correlate expression levels of CD8A (a marker indicative of CD8^+^ T cells) and VCAN across subtypes of breast cancer. Interestingly, CD8A and VCAN were nearly exclusive to one another, as revealed by the clustering of expression values across each subtype (Figure 4e).

### 3.6. VCAN Status Predicts Epithelial Infiltration of CD8^+^ T Cells

Recent reports have suggested that the location of cytotoxic T cells (intra-stromal vs. intra-epithelial) plays an important role in determining the prognostic relevance of these cells [37,38]. Therefore, these cells were stratified into either stromal or epithelial based on the tumor compartment in which the CD8^+^ T cells were localized (Figure 4f). The average epithelial CD8^+^ T cells/HPF in ER− breast cancer was double that of ER+ breast cancer (6.3 vs. 13.5, *p* = 0.058; Figure 4g). Intriguingly, the ER+ and ER− breast cancers had similar numbers of stromal CD8^+^ T cells (Figure 4h).

Despite the low prevalence of VCAN-undetectable tumors within the ER+ cohort, there was a trend toward more CD8^+^ T cells within the epithelial compartment of VCAN-undetectable cancers compared to VCAN-detectable tumors (*p* = 0.065; Supplementary Figure S2). Similarly, ER− cancers with undetectable VCAN contained significantly more intraepithelial CD8^+^ T cells (6.5 vs. 37.1, *p* = 0.022). With regard to the stromal CD8^+^ T cell counts across these subtypes, ER+ cancers demonstrated no difference based on VCAN status, whereas ER− cancers actually contained fewer stromal CD8^+^ T cells/HPF in VCAN-undetectable disease (*p* = 0.03; Supplementary Figure S2).

Upon including the PR and HER2 status of these cancers, it was found that TNBC demonstrated significantly more epithelial CD8^+^ T cells/HPF than ER+/PR+/HER2− tumors (*p* = 0.013; Figure 4i). However, there was little difference between these subgroups with regard to their numbers of stromal CD8^+^ T cells (Figure 4j). There was a trend towards more epithelial CD8^+^ T cells in VCAN-undetectable tumors in both ER+/PR+/HER2− (*p* = 0.08) and TNBC (*p* = 0.07; Figure 4k). The numbers of stromal CD8^+^ T cells actually decreased in TNBC in the absence of VCAN accumulation (*p* = 0.03; Figure 4l). To investigate whether our TMA cores were truly representative of CD8^+^ T cell localization in these tumors, full-tissue slides were stained for CD8 and evaluated by counting epithelial CD8^+^ T cells across five HPFs. The epithelial localization of CD8^+^ T cells was found to be consistent in the TMA cores when compared to full tissue sections across 32 patients (Supplementary Figure S3).

We hypothesize that stromal VCAN may sequester CD8^+^ T cells within the stromal compartment of tumors, preventing their invasion into epithelial regions and direct interaction with cancer cells. To address this question, we calculated the percentage of epithelial CD8^+^ T cells (epithelial/(epithelial + stromal)) across each of our ER/PR/HER2 subgroups, with little difference among the groups (Figure 4m). Upon separating these cancers by the detection of VCAN, it was found that TNBC demonstrated a statistically significant increase in the number epithelial % of CD8^+^ T cells in the absence of VCAN (*p* = 0.008); other subtypes demonstrated similar trends (Figure 4n).

To demonstrate the correlation of VCAN with cancer-associated fibroblast (CAF) associated genes in breast cancer, we conducted gene expression analyses across the TCGA PanCancer database for invasive breast carcinoma. It was found that VCAN expression clusters strongly with genes typically associated with stromal CAFs, such as COL1A1, FAP, PDGFRB, PDPN, and ACTA2 (Figure 4o). Additionally, expression of CD8A tended to be inversely related with VCAN and these other genes, suggesting that high expression of VCAN and the presence of fibroblasts may be exclusive of a robust epithelial infiltration of CD8A-expressing lymphocytes (likely CD8^+^ TILs).

### 3.7. VCAN Proteolysis in Breast Cancer

**Figure 5:**
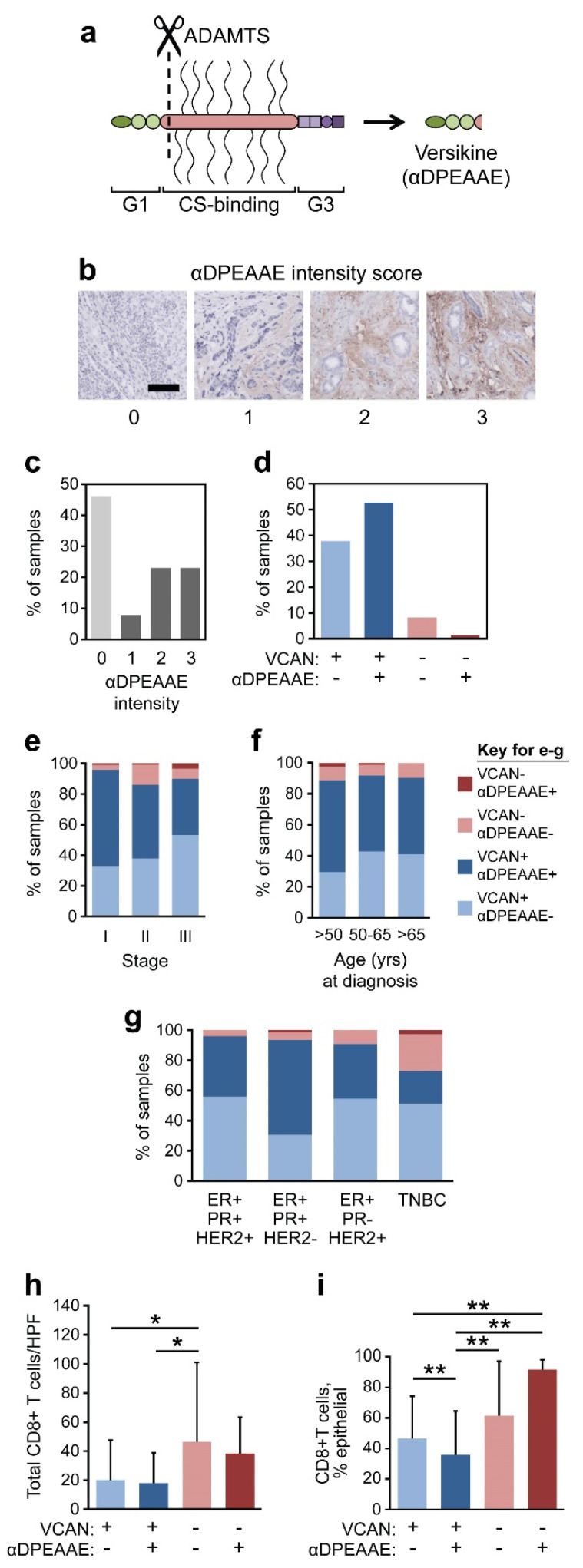
VCAN proteolysis predicts epithelial infiltration by CD8^+^ T cells. (**a**) Cartoon depiction of VCAN proteolysis via ADAMTS proteases. (**b**) Representative images of αDPEAAE binning intensities. The scale bar is 100 µM. (**c**) Distribution of patients based on αDPEAAE intensities. (**d**) Distribution of patients based on presence of VCAN and αDPEAAE. (+) indicates detectable, (−) indicates undetectable. (**e**–**g**) Distribution of patients across stage, age, and ER/PR/HER2 status. (**h**,**i**) Average total or epithelial % of CD8^+^ T cells according to VCAN/αDPEAAE status. Statistical significance was determined using Wilcoxon rank-sum tests. * *p* < 0.05; ** *p* < 0.01.

Next, we investigated the proteolysis of VCAN, an event predicted to release a bioactive fragment (matrikine), versikine (Vkine) [35]. Vkine is the 441-aa N-terminal fragment of the VCAN V1 isoform, which is released upon proteolytic cleavage by members of the ADAMTS (A Disintegrin And Metalloproteinase with a ThromboSpondin type 1 motif) family of proteases [39] (Figure 5a). Quantification of VCAN proteolysis was restricted to the tumor stroma, and it relies on the detection of the neoepitope αDPEAAE, which is formed upon proteolytic cleavage by ADAMTS proteases [40]. We had previously demonstrated that CRC tumors with both low total VCAN and high αDPEAAE staining intensity (termed VCAN proteolytic predominant, or VPP) demonstrated the greatest CD8^+^ T cell infiltration [17], and we discovered that recombinant Vkine has independent immunostimulatory activity in vitro [16,17]. Similar to VCAN accumulation, we quantified VCAN proteolysis using a binning system based on αDPEAAE intensity (Figure 5b). Of the 230 breast cancers analyzed, 45.9% did not demonstrate robust VCAN proteolysis in the stroma (0–1), while 46.3% had abundant VCAN proteolysis (2–3; Figure 5c).

Next, the cancers were categorized based on both the detection of VCAN and αDPEAAE. Due to our findings correlating biological significance in breast cancer with only the detectable/undetectable status of VCAN, we assessed VCAN proteolysis utilizing the detectable/undetectable designation for both VCAN and αDPEAAE (Figure 5d). Though rare, 1.3% of all patients were both VCAN-undetectable and αDPEAAE-detectable, and these were distributed across each stage and age group of these patients (Figure 5e,f). There was no significant difference in VCAN proteolysis across ER/PR/HER2 status (Figure 5g). Total CD8^+^ cells were significantly greater in VCAN-undetectable tumors compared to VCAN-detectable tumors regardless of the detection of αDPEAAE (Figure 5h). However, VCAN-undetectable/αDPEAAE-detectable tumors demonstrated 92% of CD8^+^ T cells penetrating the epithelial portion of the tumor, much greater than any of the other subgroups albeit without statistical significance from VCAN-undetectable/αDPEAAE-undetectable tumors (Figure 5i).

**Figure 6:**
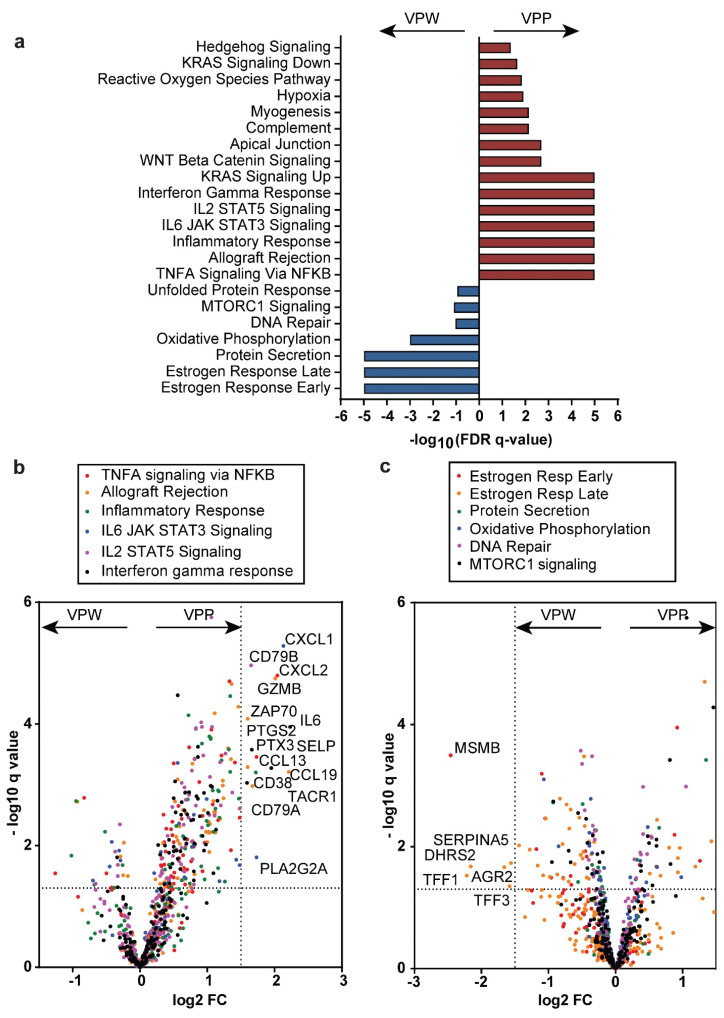
VCAN proteolysis is associated with immune infiltration pathways. (**a**) Top differentially enriched Hallmark gene sets identified from gene set enrichment analysis of VPP (VCAN^hi^ ADAMTS4^hi^ TIMP3^intermediate/low^) and VPW (VCAN^hi^ ADAMTS4^intermediate/low^ TIMP3^hi^) cohorts from the TCGA PanCancer Atlas Breast Invasive Carcinoma dataset. (**b**) Volcano plot of differential expression of genes in the top gene sets enriched in the VPP cohort. (**c**) Volcano plot of differential expression of genes in the top gene sets enriched in the VPW cohort. Dashed lines in (**b**,**c**) correspond to a log2 fold change of ±1.5 and a q-value of 0.05.

To expand this analysis, an RNA-based signature was developed, as outlined in the methods to identify cases likely to have the VCAN proteolytic predominant phenotype (VPP) or a VCAN proteolytic weak phenotype (VPW). The Breast Invasive Carcinoma TCGA PanCancer Atlas dataset was used to identify 48 VPP cases and 66 VPW cases based on the expression of *VCAN*, *ADAMTS4*, and *TIMP3*. GSEA was then performed by comparing these two cohorts. The VPP cohort was found to be enriched for pathways consistent with immune infiltration, including inflammatory response, allograft rejection, TNFα signaling, and interferon-gamma response (Figure 6a). The differentially expressed genes in the significantly altered pathways are presented in Figure 6b,c. In the VPP cancers, significant increases were observed in *CXCL1*, *CXCL2*, *GZMB*, *IL6*, *CCL13*, and *CCL19*, among others.

## 4. Discussion

Anti-PD1 immunotherapy is now a standard option in combination with chemotherapy for some patients with breast cancer, as outlined above [2,3,4,5,6]. It is only a subset of patients that actually respond, and the current biomarkers, including PD-L1 expression, fail to identify those patients most likely to respond. New biomarkers are needed to further aid in patient selection for these therapies.

VCAN has demonstrated relevance over several “hallmarks of cancer” and has been implicated in modulating both cancer cell-intrinsic and microenvironment properties in breast cancer [41,42]. Studies have demonstrated increased VCAN accumulation in breast tumors compared to normal breast tissue [43,44]. Our work corroborated these findings by showing that the vast majority of breast cancers have significant VCAN accumulation (Figure 1). This upregulation of VCAN has been associated with more aggressive disease [45] and poorer outcomes [21] for breast cancer patients.

Our prior work and other studies have highlighted the immunosuppressive activities of VCAN in solid tumors, specifically the connection between VCAN-enriched tumors and low infiltration of CD8^+^ T cells [16,17,46,47], and recruitment/activation of immunosuppressive myeloid cells [48,49]. Recent reports have discovered that VCAN-high breast cancers have a greater infiltration of tumor-associated macrophages, a critical piece of the immunosuppressive landscape of many solid tumors [20]. Additionally, in triple-negative breast cancer, an association between VCAN and the localization of CD8^+^ T cells to the tumor stroma was recently reported, and the specific chondroitin sulfate–glycosaminoglycan (CS-GAG) sulfation patterning is important for regulating T cell trafficking [47].

Predicting the infiltration of CD8^+^ T cells within solid tumors has been shown to identify patients more likely to respond to immune checkpoint inhibitors [50,51,52]. Whereas previous reports have demonstrated a difference in total CD8^+^ T cells in breast cancers across stage [53], age and menopause status [8,54,55], our findings indicate that a VCAN-status predicts more total CD8^+^ T cells across each of these clinical groups (Figure 3; Supplementary Figure S1). CD8^+^ T cell numbers have been reported to be greater in patients with negative hormonal status [56,57,58,59,60]. While we found greater CD8^+^ T cells across TNBC cancers compared to the ER+/PR+ cancers at large (Figure 4c), a lack of VCAN identified patients with a similar presence of CD8^+^ T cells regardless of ER/PR status (Figure 4d). Therefore, the higher average count of CD8^+^ T cells in TNBC can be partially attributed to the greater proportion of VCAN-undetectable TNBC patients compared to other hormone-receptor subtypes (Figure 1g). Based on these findings, VCAN detection should be explored as a biomarker for all breast cancer patients receiving immunotherapy and chemotherapy, including the neoadjuvant and metastatic settings [2,3,4,5,6].

Beyond VCAN accumulation leading to an excluded TME, VCAN proteolysis by ADAMTS proteases releases Vkine, a matrikine with immune stimulatory properties countering the immunoregulatory functions of VCAN [20,40,47]. These immunostimulatory properties include the increased/enhanced maturation, abundance, and activation of stimulatory type 1 conventional dendritic cells. Here, we demonstrate a trend for the VPP phenotype (low VCAN and high Vkine expression) to correlate with enhanced CD8^+^ T cell infiltration into the epithelial compartment, which is consistent with the immunostimulatory role of Vkine. The VPP phenotype deserves evaluation in a larger dataset as a clinical predictor of CD8^+^ T cell infiltration and immunotherapy response beyond the accumulation of VCAN as a biomarker for immune exclusion and immunotherapy resistance.

Our findings in this work further solidify the relationship between immunomodulatory VCAN accumulation and the lack of CD8^+^ T cells in many solid tumors. Many questions remain regarding the mechanisms that drive VCAN accumulation and the subsequent exclusion of tumor-reactive CD8^+^ T cells in breast cancer, and answering these questions will build on the findings presented here. Additionally, due to the robust correlation between high VCAN proteolysis and intraepithelial localization of CD8^+^ T cells, it will be necessary to understand the influence of Vkine and other VCAN-matrikines on the immune system. Investigating the utility of VCAN testing as a biomarker of immunotherapeutic response in future studies is warranted and achievable based on these studies, and identifying more accurate biomarkers in this space could expand therapeutic options and provide clinical benefit to thousands of breast cancer patients.

## 5. Conclusions

In this work, we have demonstrated a strong correlation between VCAN abundance and proteolysis and infiltration of CD8^+^ T cells. Therefore, VCAN is a promising candidate for a biomarker for immunotherapy response and should be investigated further for its predictive potential. Interestingly, our work identified that the association between VCAN and immune infiltration is consistent across breast cancer subtypes, indicating a potential to identify patients that may derive benefit from immunotherapy outside of the already approved TNBC subtype.

## Data Availability

Data are available upon email request to the corresponding author.

## Supplementary Materials

The following supporting information can be downloaded at: https://www.mdpi.com/article/10.3390/cancers17091435/s1, Supplementary Figure S1: Total CD8^+^ T cells within the breast tumor based on menopausal status. Supplementary Figure S2: Epithelial and stromal CD8^+^ T cells in ER+ and ER− breast cancer samples. Supplementary Figure S3: Correlation of VCAN presence with CD8^+^ T cell counts comparing TMA cores and full breast cancer specimen slides.
