# Supplementary material for: Stromal Versican Accumulation and Proteolysis Regulate the Infiltration of CD8+ T Cells in Breast Cancer
Source: Cancers (Basel). 2025 Apr 25;17(9):1435. doi: 10.3390/cancers17091435 (PMC12070914; doi:10.3390/cancers17091435)
Supplement: Supplementary file 1 [file cancers-17-01435-s001.zip › cancers-3505048-supplementary.pdf]

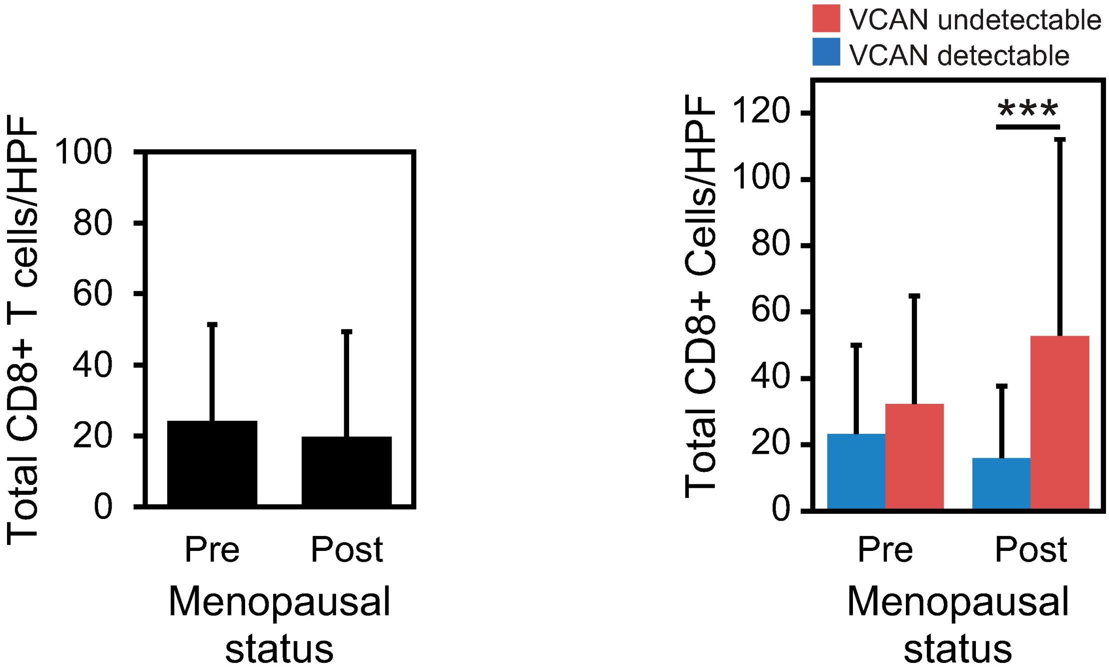

Supplementary Figure S1: Total CD8⁺ T cells within the breast tumor based on menopausal status.

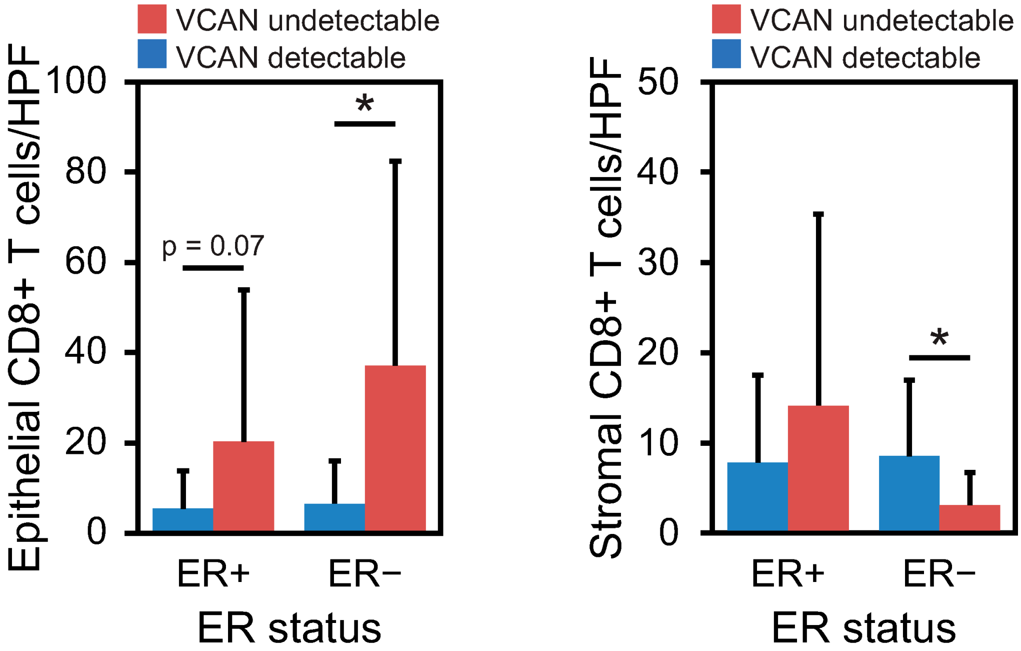

Supplementary Figure S2: Epithelial and stromal CD8⁺ T cells in ER+ and ER− breast cancer samples.

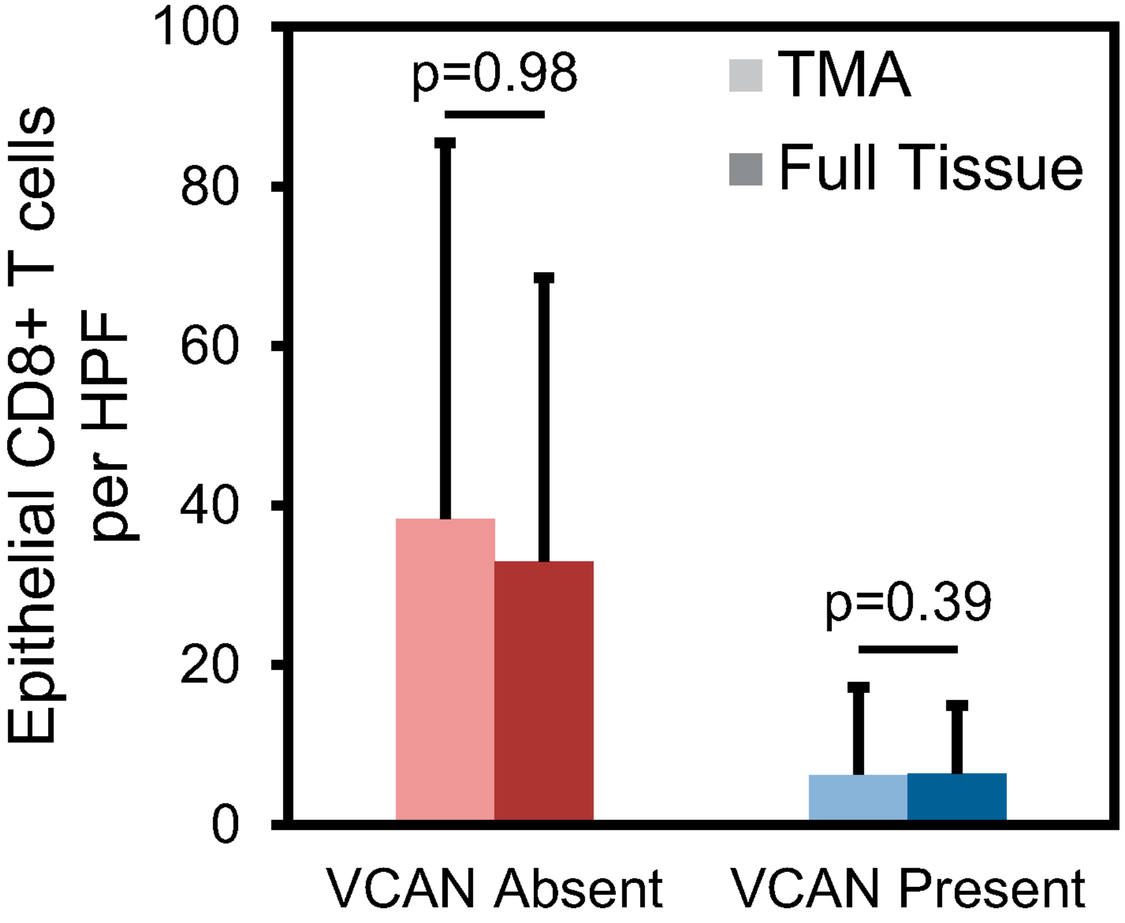

Supplementary Figure S3: Correlation of VCAN presence with $CD8^{+}$ T cell counts comparing TMA cores and full breast cancer specimen slides.
